# Methylation analysis of the *glypican 3* gene in embryonal tumours

**DOI:** 10.1038/sj.bjc.6601716

**Published:** 2004-03-23

**Authors:** G Boily, Z Saikali, D Sinnett

**Affiliations:** 1 Division of Hematology-oncology, Charles-Bruneau Cancer Center, Research Center, Sainte-Justine Hospital, 3175 chemin de la Côte-Sainte-Catherine, Montreal, Quebec, Canada H3T 1C5; 2 Department of Pediatrics, University of Montreal, 3175 chemin de la Côte-Sainte-Catherine, Montreal, Quebec, Canada H3T 1C5

**Keywords:** *GPC3*, embryonal tumours, transcriptional regulation, methylation, X chromosome

## Abstract

We have previously shown that the *glypican 3* (*GPC3*) gene was expressed in neuroblastoma (NB) and Wilms' tumour (WT), two embryonal tumours. *GPC3* is an X-linked gene that has its peak expression during development and that is downregulated in all investigated tissues after birth. *GPC3* expression could be involved in the aetiology of embryonal tumours such as NB and WT. Methylation is known to play a role in gene silencing, notably in chromosome X inactivation. Southern blot- and PCR-based methylation assays were used to assess the methylation status of the *GPC3* promoter on genomic DNA from both normal and embryonal tumour cells. In normal cells, the promoter was not methylated in males and partially methylated in females. Our results suggest that DNA methylation of the promoter region is not essential for the transcriptional repression of the *GPC3* gene and that the methylation observed in females is probably linked to the inactive X chromosome. In tumour samples, methylation abnormalities have been found exclusively in female NB samples (loss of methylation) and mainly in male WT samples (gain of methylation). Overall, methylation did not significantly correlate with the expression status of *GPC3*. Although promoter methylation is likely to affect the expression status of the gene, our results suggest that the deregulation of *GPC3* transcriptional expression seen in NB and WT involves other regulatory levels.

*Glypican 3* (*GPC3*) gene has been shown to be expressed in neuroblastoma (NB) and Wilms' tumour (WT), two embryonal tumours (Saikali and Sinnett, 2000; Toretsky *et al*, 2001). This gene is expressed in a tissue-specific manner and has its peak expression during development (Filmus *et al*, 1988; Hsu *et al*, 1997; Pellegrini *et al*, 1998). After birth, *GPC3* is downregulated in all investigated tissues (Filmus *et al*, 1988; Hsu *et al*, 1997; Pellegrini *et al*, 1998). *GPC3* is located at chromosome Xq26.1 and spans more than 500 kb (Pilia *et al*, 1996; Huber *et al*, 1997; Shen *et al*, 1997). The gene product is a heparan sulphate proteoglycan located on the cell surface and attached to the cellular membrane by a glycosyl-phosphatidyl inositol anchor (Pilia *et al*, 1996). The role of this protein is not yet exactly known. Many studies suggest that GPC3 is a negative cellular growth regulator (Pilia *et al*, 1996; Weksberg *et al*, 1996; Cano-Gauci *et al*, 1999; Lin *et al*, 1999; Murthy *et al*, 2000; Xiang *et al*, 2001), one of the most compelling evidence being that a germline mutation of the gene causes the Simpson–Golabi–Behmel overgrowth syndrome (Pilia *et al*, 1996) and that *Gpc3* knockout mice partly recapitulate the syndrome (Cano-Gauci *et al*, 1999). On the other hand, *GPC3* has been shown to be overexpressed in hepatocellular carcinomas (Hsu *et al*, 1997; Toretsky *et al*, 2001; Zhu *et al*, 2001; Midorikawa *et al*, 2003) and to be associated with advanced stages as well as with the invasive potential of this cancer (Hsu *et al*, 1997). Moreover, colorectal carcinoma-associated liver metastases express *GPC*3 significantly more than primary tumours (Lage *et al*, 1998). These data suggest that GPC3 is regulating different growth and survival factors in a cell-dependent manner (Filmus, 2001).

The mechanisms regulating the transcription of *GPC3* are of particular interest to understand the altered expression of *GPC3* in cancer cells. *GPC3* has been shown to be overexpressed preferentially in female as compared to male hepatocellular carcinomas (females, 95% and males, 67%) (Hsu *et al*, 1997). As the gene is located on the X chromosome and DNA methylation is implicated in chromosome X inactivation (Monk, 1986), this observation raises the possibility that a loss of methylation could be implicated in the overexpression of *GPC3* in some cancer forms. Hypermethylation of the *GPC3* promoter associated with gene silencing has been observed in certain adult cancers (Huber *et al*, 1999; Lin *et al*, 1999; Murthy *et al*, 2000; Xiang *et al*, 2001). Owing to the potential involvement of GPC3 expression in the aetiology of embryonal tumours, we tested the methylation status of the *GPC3* promoter in DNA samples derived from both normal and embryonal tumour cells.

## MATERIALS AND METHODS

### DNA samples

In this study, we used genomic DNA from the following sources: 14 peripheral blood samples (obtained from volunteer healthy donors at the Sainte-Justine Hospital, Montreal, Canada); 11 placenta samples (DNA was obtained from C Deal); NB cell lines SK-N-AS, SK-N-DZ, SK-N-FI, IMR-32, SK-N-SH (obtained from ATCC, Manassas, VA, USA), NBL-S (obtained from GM Brodeur), SJNB-1, SJNB-7, SJNB-10 (obtained from T Look); primary NB and WT specimens (obtained from patients treated at Sainte-Justine Hospital); and two peripheral blood samples from Turner syndrome patients (DNA and karyotypes were obtained from C Deal). This study was approved by our Institutional Review Board.

### Methylation assays

#### Cytosine methylation assay

**Figure 1:**
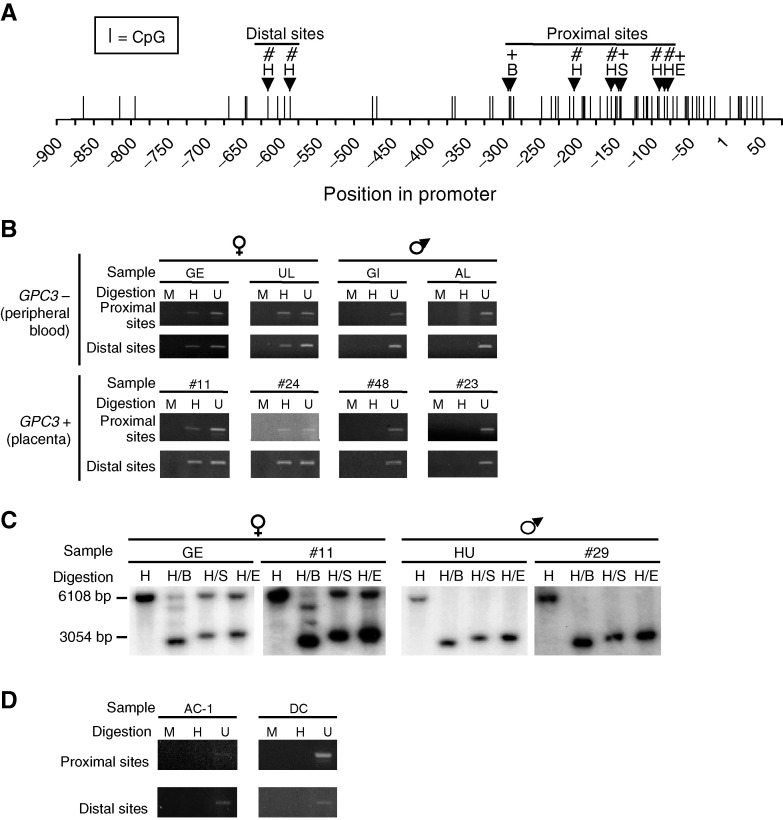
Methylation analysis of the *GPC3* promoter in nontumoural samples. (**A**) CpG dinucleotides positions in the *GPC3* promoter region. The methylation status of 11 of these CpG sites was determined either by the PCR-based methylation assay (#) or by the Southern blot-based methylation assay (+) using methyl-sensitive restriction endonucleases *Hpa*II (H), *Sac*II (S), *Eag*I (E), and *Bss*HII (B). *Hpa*II contains one CpG site, whereas *Sac*II, *Eag*I and *Bss*HII contain two CpG sites each. The distal and proximal sites were amplified in distinct PCRs. (**B** and **C**) Representative results of the methylation analysis in normal peripheral blood and placental DNA samples obtained by the PCR-based (**B**) and Southern blot-based (**C**) methylation assays. See Table 1 for details concerning the samples. (**D**) PCR-based methylation assay performed on DNA samples from two individuals (AC-1 and DC) affected by the Turner syndrome. Digestions: (**B** and **D**) H, *Hpa*II; M, *Msp*I; U, undigested; (**C**) H, *Hind*III; B, *Bss*HII; S, *Sac*II and E, *Eag*I. GPC3+, expression of GPC3; GPC3−, no expression of GPC3.

The promoter region of GPC3 contains a CpG island (Figure 1A

) (Huber *et al*, 1997). Several attempts to apply bisulphite protocols (e.g. Cote *et al*, 1998) in order to assess the methylation status of this promoter region were unsuccessful (data not shown). This apparent resistance to deamination is not unique to GPC3 (e.g. Bearzatto *et al*, 2000) and could be explained by the high CG content (Harrison *et al*, 1998). We decided to use two alternative approaches (see below).

#### PCR-based methylation assay

For the PCR-based method, 200 ng of genomic DNA and 200 fg of a control plasmidic DNA construct (pBlueScript vector with, as an insert, a 102 bp HPRT gene fragment containing three *Hpa*II/*Msp*I sites within position 1256–1357, Accession number M26434) were digested with 100 U of *Hpa*II or *Msp*I (New England Biolabs, Beverly, MA, USA) for 16 h (see Benachenhou *et al*, 1998). The cleavage at the six *Hpa*II/*Msp*I sites located within the 700 bp upstream of the *GPC3* transcription initiation site was examined by the means of two PCR reactions (one for the two distal sites and one for the four proximal sites; Figure 1A). Polymerase chain reactions were performed in a total volume of 20 *μ*l containing 1 *μ*l of the *Hpa*II or *Msp*I digestion reactions (10 ng of genomic DNA), 1 × of ‘GC Genomic PCR Reaction Buffer’ (Clontech, Palo Alto, CA, USA), 1.1 mM Mg(OAc)_2_, 200 *μ*M of each of the four dNTPs, 1 M ‘GC-Melt’ (Clontech, Palo Alto, CA, USA), 0.4 *μ*M of each primer (proximal sites: B2 (ACGTGCTGCTACCCAGCCGCTGCA) and L2 (GGAACTTCTCCCAGAGCCAGTCAGAGCG); distal sites: E2 (CCGCTCATTGGCCTACAGCCTGGAGGGC) and J2 (TATTCAAAGGTGAGGCAGGCTGTGAAAAGC)) and 1 × of ‘Advantage GC Genomic Polymerase Mix’ (Clontech, Palo Alto, CA, USA). Polymerase chain reactions for the proximal sites were performed for one cycle of 95°C for 1 min, followed by 38 cycles of 95°C for 45 s, and 74°C for 2 min, followed by one cycle of 74°C for 10 min. PCR reactions for the distal sites were performed for one cycle of 95°C for 1 min, followed by 28 cycles of 95°C for 30 s, and 68°C for 2 min, followed by one cycle of 68°C for 10 min. Complete cleavage was verified by a PCR amplification of the control construct insert with 1 *μ*l of the *Hpa*II or *Msp*I digestion reactions (10 fg plasmid DNA) under standard conditions.

#### Southern blot-based methylation assay

Genomic DNA was digested with *Hind*III, either alone or with methyl-sensitive restriction endonucleases *Eag*I, *Sac*II or *Bss*HII. The digestion products were electrophoresed on agarose gels and transferred onto Hybond N+ nylon membranes (Amersham Pharmacia Biotech, Baie d'Urfé, Canada). The membranes were hybridised with a radiolabelled *GPC3* promoter-specific PCR product (positions −969 to −346, Figure 1A). In this assay, a 6.1 kb fragment is expected when the investigated sites are fully methylated, whereas a fragment of about 3 kb should be obtained when the sites are not methylated.

### Statistical analysis

In order to evaluate whether methylation abnormalities was significantly more frequent in female or male tumour samples, the Fisher's exact test was used. A methylation profile was considered abnormal when it was different from the methylation profile observed in apparently normal samples (peripheral blood and placentas) of the same gender.

## RESULTS

In all, 11 CpG sites located in the promoter of the *GPC3* gene have been tested for methylation using methyl-sensitive restriction endonuclease assays (Figure 1A). Six of them were located within *Hpa*II sites and were tested by the PCR-based methylation assay along with undigested and methyl-insensitive *Msp*I-digested samples as controls. The five others were located within *Eag*I, *Sac*II and *Bss*HII methyl-sensitive restriction sites and were investigated with the Southern blot-based methylation assay.

These sites were first tested in DNA samples derived from normal cells including 14 peripheral blood samples, known not to express *GPC3* (*GPC3*−) (Hsu *et al*, 1997), and 11 placenta samples, which strongly express *GPC3* (*GPC3*+) (Hsu *et al*, 1997). We found that, independent of the expression status, methylation correlated with the gender: females presented a partial methylation, whereas males had no methylation (Figure 1B and C). These results suggest that methylation is not essential for the repression of the *GPC3* gene, since *GPC3* nonexpressing male samples are not methylated at the studied sites (Table 1

**Table 1:** Summary of the *GPC3* promoter methylation data of normal cells

			**Methylation status**				
			**PCR-based assay^a^**		**Southern blot-based assay^b^**		
**Sample**	**Origin**	**Gender^c^**	**Proximal sites**	**Distal sites**	***Eag*I**	***Sac*II**	***Bss*HII**
GE	Periph. blood	F	+	+	+/−	+/−	+/−
CA	Periph. blood	F	+	+	+/−	+/−	+/−
UL	Periph. blood	F	+	+	+/−	+/−	+/−
CP41	Periph. blood	F	+	+	ND	ND	ND
CP42	Periph. blood	F	+	+	ND	ND	ND
CP43	Periph. blood	F	+	+	ND	ND	ND
GI	Periph. blood	M	−	−	−	−	−
AL	Periph. blood	M	−	−	−	−	−
HU	Periph. blood	M	−	−	−	−	−
PED 93	Periph. blood	M	−	−	ND	ND	ND
CP111	Periph. blood	M	−	−	ND	ND	ND
CP112	Periph. blood	M	−	−	ND	ND	ND
CP113	Periph. blood	M	−	−	ND	ND	ND
CP114	Periph. blood	M	−	−	ND	ND	ND
#11	Placenta	F	+	+	+/−	+/−	+/−
#24	Placenta	F	+	+	+/−	+/−	+/−
#25	Placenta	F	−	+	+/−	+/−	+/−
#26	Placenta	F	+	+	ND	ND	ND
#27	Placenta	F	+	+	ND	ND	ND
#28	Placenta	F	+	+	ND	ND	ND
#20	Placenta	M	−	−	ND	ND	ND
#23	Placenta	M	−	−	ND	ND	ND
#29	Placenta	M	−	−	−	−	−
#32	Placenta	M	+	+	ND	+/−	+/−
#48	Placenta	M	−	−	−	−	−

*GPC3*=glypican 3; PCR=polymerase chain reaction.

a +=methylated; −=not methylated.

b +=methylation signal; −=nonmethylation signal; +/−=both methylation and nonmethylation signals; ND=not determined.

c F=female; M=male.

). Southern blot methylation *vs* nonmethylation signal intensities presented a ratio of approximately 1 : 1 in females, indicating the presence of methylation in about half of the DNA molecules (Figure 1C). This suggests that the methylation detected in females could be linked to the inactive X chromosome. Male sample #32 *GPC3* promoter has been shown to be partially methylated as opposed to other male samples (Table 1). Sex determination assay and X chromosome microsatellite amplification (DXS102, DXS538 and DXS981) showed that this sample has a Y chromosome and only one X chromosome (data not shown). This suggests that the partial methylation seen in sample #32 reflects cell heterogeneity for *GPC3* promoter methylation. PCR-based methylation assay on female sample #25 showed that at least one of the proximal sites was not methylated (Table 1). However, the Southern blot-based assay methylation profile of this sample was similar to that of the other female samples, suggesting that the *GPC3* promoter is methylated but not at every site.

In order to test the hypothesis that *GPC3* promoter methylation in females is linked to the inactive X chromosome, the PCR-based methylation assay was performed on peripheral blood DNA samples from two Turner syndrome patients with karyotype (45, X), having no inactive X chromosome. No methylation signal was detected (Figure 1D), supporting the hypothesis that the methylation signal detected at the *GPC3* promoter is linked to the inactive X chromosome.

**Figure 2:**
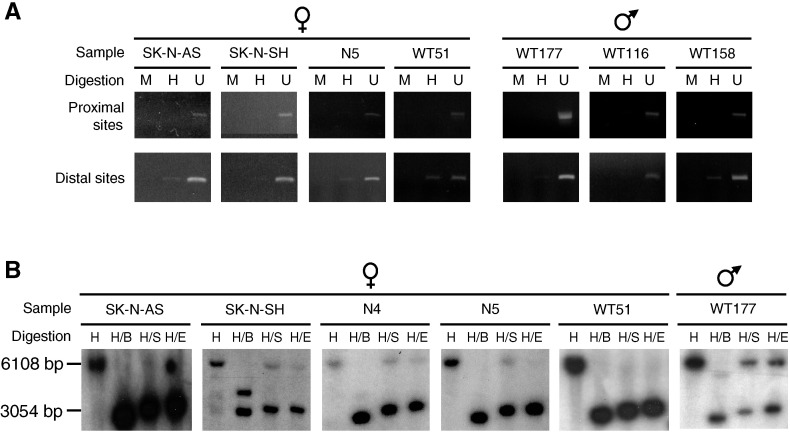
Methylation analysis of the *GPC3* promoter in tumour cell DNA samples. PCR- (**A**) and Southern blot- (**B**) based methylation assays were performed on tumour cell DNA samples from NB cell lines (SK-N-AS, SK-N-SH), primary NBs (N4, N5) and primary WTs (WT51, WT116, WT158, WT177). Only results for samples with abnormal DNA methylation patterns are shown. Digestions: (**A**) H: *Hpa*II; M: *Msp*I; U: undigested; (**B**) H: *Hind*III; B: *Bss*HII; S: *Sac*II and E: *Eag*I.

PCR- and Southern blot-based methylation assays were performed on the *GPC3* promoter of NB cell lines, primary NBs and primary WTs (Figure 2

). Overall in NB samples, four females out of six (67%) showed some loss of methylation, whereas every males had normal methylation status (Figure 2, Table 2

**Table 2:** Summary of the *GPC3* promoter methylation data of tumour cells and their *GPC3* mRNA expression status

				**Methylation status**				
				**PCR-based assay^a^**		**Southern blot-based assay^b^**		
**Sample**	**Origin**	**Gender^c^**	**Expression^d^**	**Proximal sites**	**Distal sites**	***Eag*I**	***Sac*II**	***Bss*HII**
SK-N-AS	NB cell line	F	+++	−	tr	−	−	−
SK-N-SH	NB cell line	F	++	−	tr	tr/−	tr/−	+/−
SK-N-DZ	NB cell line	F	−	+	+	+/−	+/−	+/−
SJNB-1	NB cell line	M	++	−	−	−	−	−
SJNB-7	NB cell line	M	+++	−	−	−	−	−
SJNB-10	NB cell line	M	+++	−	−	ND	ND	ND
NBL-S	NB cell line	M	++	−	−	ND	ND	ND
IMR-32	NB cell line	M	++	−	−	ND	ND	ND
SK-N-FI	NB cell line	M	−	−	−	−	−	−
NB4	Primary NB	F	+	+	+	tr/−	tr/−	tr/−
NB5	Primary NB	F	+	−	tr	−	tr/−	tr/−
NB8	Primary NB	F	ND	+	+	ND	ND	ND
NB11	Primary NB	M	−	−	−	−	−	−
NB13	Primary NB	M	+	−	−	−	−	−
NB193	Primary NB	M	ND	−	−	ND	ND	ND
WT130	Primary WT	F	++	+	+	+/−	+/−	+/−
WT42	Primary WT	F	+	+	+	+/−	+/−	+/−
WT51	Primary WT	F	++	−	+	−	−	−
WT106	Primary WT	F	+	+	ND	ND	ND	ND
WT40	Primary WT	M	+++	−	−	−	−	−
WT177	Primary WT	M	++	−	tr	+/−	+/−	+/−
WT116	Primary WT	M	+	tr	−	ND	ND	ND
WT158	Primary WT	M	ND	tr	+	ND	ND	ND

*GPC3*=glypican 3; PCR=polymerase chain reaction; NB**=**neuroblastoma; WT=Wilms' tumour.

a +=methylated; −=not methylated.

b +=methylation signal; −=nonmethylation signal; tr=traces; +/−=both methylation and nonmethylation signals; tr/−=both traces of methylation signal and nonmethylation signal; ND=not determined.

c F=female; M=male.

d Transcriptional expression: −=no expression; +=weak expression; ++=moderate expression; +++=strong expression. The analysis of the expression levels was reported in Saikali *et al* (2000) and was based on the visual inspection of all the Northern blots or semiquantitative RT–PCR assays.

), suggesting that methylation abnormalities are predominantly found in females (Fisher's test: *P*=0.011). Methylation analysis of the *GPC3* promoter in WT samples also revealed abnormalities when compared to the normal cells. One female out of four (WT51) presented a loss of methylation and three males out of four (75%) showed partial methylation (Figure 2, Table 2). Therefore, in contrast to NB, in WT samples, methylation abnormalities seem to be more frequent in males than in females. However, more samples need to be investigated to confirm this trend (Fisher's test: *P*=0.243).

In most cases, as in normal cells, the methylation pattern of the *GPC3* promoter at the investigated sites is not correlated with the expression status (Table 2). However, in female NB samples, loss of methylation correlates with the expression of *GPC3* (Table 2), raising the possibility that loss of methylation of the inactive X chromosome could lead to the transcriptional activation of the linked *GPC3* allele. To test this hypothesis, the cell lines SK-N-DZ (normal methylation pattern, *GPC3*−; Table 2) and SK-N-SH (loss of methylation, *GPC3*+; Table 2) were treated with 0.5, 1 and 5 *μ*M of demethylating agent 5-aza-deoxycytidine (5-aza-dC). Southern blot-based methylation assay revealed that no demethylation was achieved, and transcriptional expression was similar to the nontreated controls as evaluated by Northern blot analysis (data not shown), even in cells treated at highly toxic concentration of 5-aza-dC (data not shown).

## DISCUSSION

In this report, we showed in peripheral blood (GPC3−) and placental (GPC3+) cells that the *GPC3* promoter methylation status at the investigated CpG sites was correlated with gender rather than the expression status, male samples being unmethylated and female samples being partially methylated. These observations are consistent with those of another methylation analysis of the *GPC3* promoter performed on leucocyte DNA samples (Huber *et al*, 1999).

These results indicate that methylation at these sites is not essential for the repression of *GPC3*. However, we cannot exclude the possibility that the CpG sites investigated are not critical for the repression of the gene. The Southern blot-based methylation assay that allows a quantitative analysis of methylation taken together with the analysis of females with Turner syndrome support the hypothesis that the methylation observed in females is associated with the inactive X chromosome. In this regard, Huber *et al* (1999) have reported a complete methylation of the *GPC3* promoter in somatic hybrid hamster–human cells containing only the human inactive X chromosome. These results strongly suggest that the *GPC3* allele located on the inactive X chromosome is methylated, whereas the active X chromosome allele is not. The methylation on the inactive X chromosome is thought to be important for the maintenance of gene silencing (Monk, 1986).

The methylation analysis in embryonal tumours revealed methylation abnormalities particularly in female NB cells and in male WTs. These observations might result from the fact that cancer cells often present aberrant methylation, their genome being generally hypomethylated and locally hypermethylated, notably in CpG islands (Baylin *et al*, 1998; Momparler and Bovenzi, 2000; Robertson and Jones, 2000). Our study suggests that the main methylation abnormalities at the *GPC3* promoter level seems to be losses of methylation in NBs and the opposite in WTs. Do methylation abnormalities have an influence on the expression status of *GPC3*? In the embryonal tumour cells tested, as in normal cells, we failed to observe any correlation between methylation and expression of GPC3. However, it has been shown *in vitro* that the *GPC3* promoter does not activate the transcription of a reporter gene when methylated (Huber *et al*, 1999). In light of these results, it seems likely that the transcriptional activation of the *GPC3* gene requires an absence of methylation of the gene promoter, but that the absence of methylation alone does not necessarily lead to transcriptional activity. It is thus possible that the loss of methylation we observed in female NBs allows the inactive X chromosome *GPC3* allele to become transcriptionally active, eventually leading to a dosage effect in the corresponding cells. The same mechanism could also explain the preferential overexpression of *GPC3* in women affected with hepatocellular carcinomas (Hsu *et al*, 1997).

In summary, *in vivo* DNA methylation of the promoter regions does not seem to be the predominant regulatory mechanism for the GPC3 gene. Thus the apparent deregulation of the *GPC3* mRNA expression reported in embryonal tumours (Saikali and Sinnett, 2000) is likely to involve other regulatory signals.
